# Effectiveness of a smart phone app on improving immunization of children in rural Sichuan Province, China: study protocol for a paired cluster randomized controlled trial

**DOI:** 10.1186/1471-2458-14-262

**Published:** 2014-03-20

**Authors:** Li Chen, Wei Wang, Xiaozhen Du, Xiuqin Rao, Michelle Helena van Velthoven, Ruikan Yang, Lin Zhang, Jeanne Catherine Koepsell, Ye Li, Qiong Wu, Yanfeng Zhang

**Affiliations:** 1 Department of Integrated Early Childhood Development, Capital Institute of Paediatrics, No. 2 Yabao Road, Chaoyang District, Beijing 100020, P.R. China; 2 Global eHealth Unit, Department of Primary Care and Public Health, Imperial College London, London, United Kingdom; 3 Save the Children China Program, Beijing, China; 4 Save the Children China Program, Chengdu, China; 5 Save the Children, Washington DC, USA

**Keywords:** Randomized controlled trial, Immunization [MeSH], Usability, Feasibility Studies [MeSH]

## Abstract

**Background:**

Although good progress has been achieved in expanding immunization of children in China, disparities exist across different provinces. Information gaps both from the service supply and demand sides hinder timely vaccination of children in rural areas. The rapid development of mobile health technology (mHealth) provides unprecedented opportunities for improving health services and reaching underserved populations. However, there is a lack of literature that rigorously evaluates the impact of mHealth interventions on immunization coverage as well as the usability and feasibility of smart phone applications (apps). This study aims to assess the effectiveness of a smart phone-based app (Expanded Program on Immunization app, or EPI app) on improving the coverage of children’s immunization.

**Methods/Design:**

This cluster randomized trial will take place in Xuanhan County, Sichuan Province, China. Functionalities of the app include the following: to make appointments automatically, record and update children’s immunization information, generate a list of children who missed their vaccination appointments, and send health education information to village doctors. After pairing, 36 villages will be randomly allocated to the intervention arm (n = 18) and control arm (n = 18). The village doctors in the intervention arm will use the app while the village doctors in the control arm will record and manage immunization in the usual way in their catchment areas. A household survey will be used at baseline and at endline (8 months of implementation). The primary outcome is full-dose coverage and the secondary outcome is immunization coverage of the five vaccines that are included in the national Expanded Program on Immunization program as well as Hib vaccine, Rotavirus vaccine and Pneumococcal conjugate vaccine. Multidimensional evaluation of the app will also be conducted to assess usability and feasibility.

**Discussion:**

This study is the first to evaluate the effectiveness of a smart phone app for child immunization in rural China. This study will contribute to the knowledge about the usability and feasibility of a smart phone app for managing immunization in rural China and to similar populations in different settings.

**Trial registration:**

Chinese Clinical Trials Registry (ChiCTR): ChiCTR-TRC-13003960

## Background

Substantial gains in child survival over the past two decades have been achieved, but still key work is required within the context of the Millennium Development Goals (MDGs) on child survival [1]. Full-dose coverage of immunization is one of the essential child health strategies and a critical public health objective, which was included in the World Health Organization (WHO)/The United Nations Children’s Fund (UNICEF) Regional Child Survival Strategy as one of the major components of the child survival intervention package [2]. It is recognized that timely vaccination, particularly within the first six months of life, is extremely important in reducing the burden of disease preventable by vaccines [3]. WHO stated in 2013 that the estimated number of all deaths of children aged less than 5 years from vaccine-preventable diseases was 1.49 million [4].

In China, good progress has been made in introducing Bacillus Chalmette Guerin (BCG), Oral Poliomyelitis Vaccine (OPV), Diphtheria Pertussis Tetanus Combined Vaccine (DTP) and Measles Vaccine since the mid-1970s. In 1992, Hepatitis B Vaccine (HBV) was included into the national immunization program. According to the guideline issued by Ministry of Health in China, caregivers of children should go to the local eligible hospitals to register for an immunization card within one month after children’s birth. In rural areas, caregivers usually go to township hospitals or village clinics for immunization services. The township doctors record the name, birth date and other basic information of the children on their immunization cards and inject the age-appropriate vaccines according to the guideline (Additional file 1: Table S1). After that, doctors record the date for the next vaccines and make appointments with caregivers.

The implementation of Expanded Program on Immunization (EPI) has improved the national immunization coverage significantly. According to the fourth National Health Service Survey (NHSS) in 2008, around 80% of Chinese children aged 12–59 months were fully immunized (one dose of BCG and measles, three doses of DPT, HBV and OPV) [5]. However, it varied greatly among provinces, with the highest in Zhejiang (89.9%), the lowest in Chongqing (46.4%), and a rate in between these two provinces in Sichuan (70.0%). Compared to the national target set for the year 2020 (more than 95% of children fully immunized) [6], there is a big gap that needs to be closed, especially for areas that have the lowest coverage. A study in Sichuan Province aiming to evaluate the current status of the childhood immunization concluded that the proportion of missed and unqualified vaccination was higher in remote rural areas [7].

An information gap both from the service supply and demand side hinders timely vaccination of children. A study focusing on the low coverage of full dose immunization found the following reasons for failure of vaccinating children on time: 1) village doctors reported that the time interval between the two injections was too long for them to remember the exact date for next injection, which resulted in the missed or overdue vaccinations. 2) caregivers reported that they did not know where to get vaccination or which doctor was responsible for their children’s vaccination and that they did not inform them of the date for next immunization [8].

Mobile health (mHealth) may have great potential to address some of the issues and challenges related to the disparity of immunization coverage in rural China. mHealth lacks a unified definition, but can be described as supporting the practice of medicine and public health by mobile devices [9]. Though the mHealth field is still in its infancy, it can have considerable impact health outcomes by, among other methods,extending the reach of health information and services to remote populations and promoting health services [9].

Recently, smart phones have found their ways into our lives. They combine the functions of a mobile phone and PDA (Portable Digital Assistant), in addition to enabling Internet access and imaging and video functionality. Apps as an indispensable part of mHealth have obtained more and more attention. Carter et al. showed that an app was acceptable and feasible as an intervention for weight loss [10]. Whittaker et al. found a multimedia mobile phone smoking cessation program (app) was technically feasible to help the target population to quit smoking [11]. Both of these studies showed that the apps were acceptable and feasible as health interventions. However, there is a lack of literature that rigorously evaluates the impact of mHealth interventions [9,12] on childhood immunization coverage [13,14].

To improve the current status of immunization coverage in rural Sichuan Province, EPI app, a smart phone app, has been developed by Save the Children and will be implemented in two townships of Xuanhan County, Sichuan Province. The aims of this study are to evaluate the usability and feasibility of the app and to explore the effectiveness of a smart phone app on improving immunization of children in rural Sichuan Province, China.

## Methods/Design

### Design

**Figure 1:**
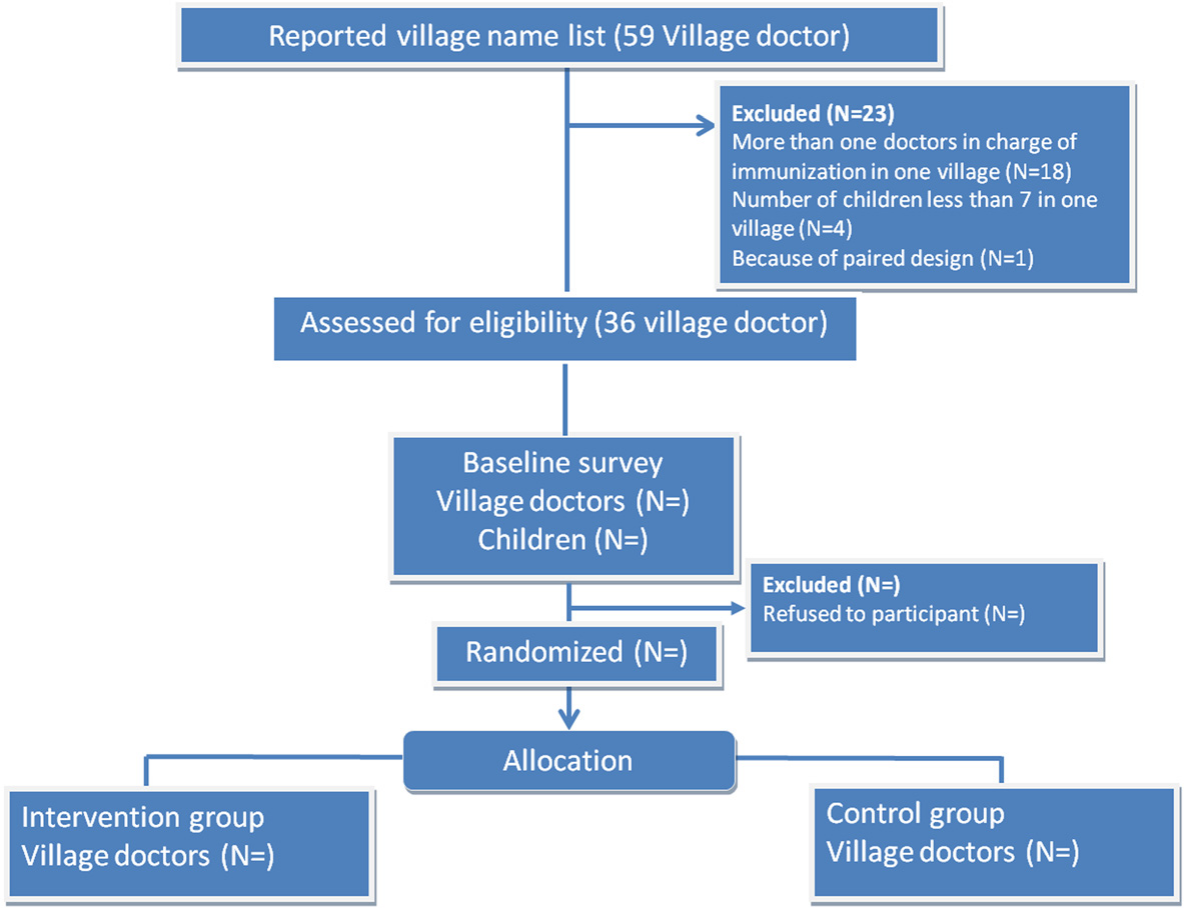
Flowchart of study.

This cluster randomized controlled trial (C-RCT) will evaluate the effectiveness of a smart phone app, the EPI app, on increasing immunization coverage of children in rural Sichuan Province (Figure 1). The village doctors will use the app to document and manage the vaccination injection records. In some villages, more than one village doctor is eligible for child vaccination and potential contamination will be introduced if we randomize at individual level (for example when a village doctor in the intervention group shares the app with another village doctor in the control group in one village). Therefore, a cluster design will be used and the unit of randomisation and analysis will be the village. The effectiveness of immunization coverage will be collected from a Maternal, Newborn and Child Health Household survey and assessed by comparing outcomes between baseline and endline (8 months after baseline).

**Figure 2:**
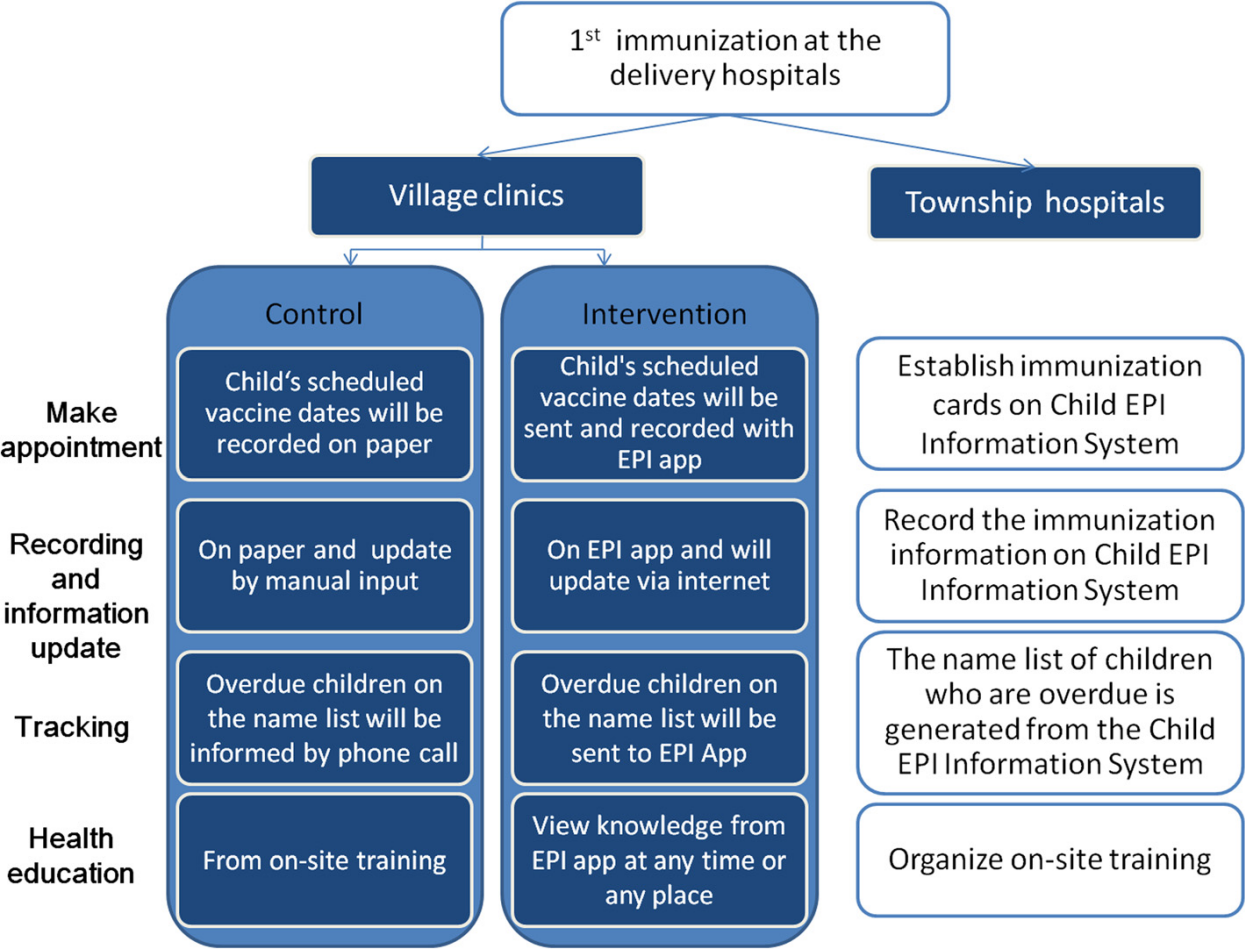
Management of immunization of children at township and village level.

The EPI app was developed by Save the Children and managed jointly by Save the Children and Xuanhan Centers of Disease Control and Prevention (CDC). Capital Institute of Pediatrics will be involved in the monitoring and evaluation process. Villages will be randomly allocated to one of two groups: the intervention group where the village doctors use a smart phone with EPI app to record and manage the vaccination and the control group where the village doctors use usual way to record and manage the vaccination (Figure 2).

### Study area

This trial will be conducted in Nanba and Xiaba Township, Xuanhan County in Dazhou City, Sichuan Province, China. Sichuan Province is located in the west part of China with an area of 486,000 km^2^ and has 21 cities and 183 counties. By the end of 2012, the total population in Sichuan Province was 80,762,000. In 2012, the annual per capita net income of rural residents was 7,001 Yuan [15]. Xuanhan County covers an area of 4,271 km^2^ and has a total population of 1,170,000. It has 55 townships and 497 villages [16]. Nanba Township has 31 villages with a total population of 100,000 and under 2 population of 1,350; Xiaba Township has 15 villages with a total population of 33,750 and under 2 population of 560.

### Intervention

#### Development of the EPI app

The Android-based EPI app was developed specifically for Xuanhan County, Sichuan Province, China by an information technology company (Cybermax, 金卫信). A smart phone app was chosen over other information communication technology solutions for the following reasons: 3G wireless networks have better coverage than broadband networks, the cost of smart phone is lower than a computer or laptop and it is easier to carry a smart phone when village doctors conduct out-reach services. The development process started with a needs assessment of village doctors in December 2012. After reaching consensus with Save the Children, a study supervision team consisting of different stakeholders including project partner PATH, local health authorities, CDCs, township hospital health workers and village doctors, and the app developer, a requirement specification of the EPI app was made early 2013. The first version of EPI app came out in September 2013 and further pilot testing and revising the app was conducted between September 2013 and March 2014.

#### Training village doctors to use EPI app and subsequent support

In January 2014, intervention village doctor each took part in a one-day training workshop on the use of EPI app. The trainers, who are the developers of the EPI app, used a variety of techniques, including interactive lectures, demonstrations and skills practice. To be judged as competent, each village doctor had to complete a skills assessment test satisfactorily.

Refresher workshops have been planned twice afterwards throughout the study. Village doctors are compensated for their travel costs and food while attending the workshops. None of the participants receive salary support or other incentives for their participation in the study. After the initial training, each village doctor in the intervention arm is provided with a 3G wireless network smart phone with the pre-installed EPI app. All the fees including phone calls, text messaging and internet access will be paid by the program. If village doctors encounter any problems when using the app during the study, they can call the township doctors for help, or if not solved by township doctors, technical support from the developer team will be provided. Moreover, a technician from the app development company will stay in our study area after the initial training to provide immediate technical support.

### Description of the smart phone app

**Figure 3:**
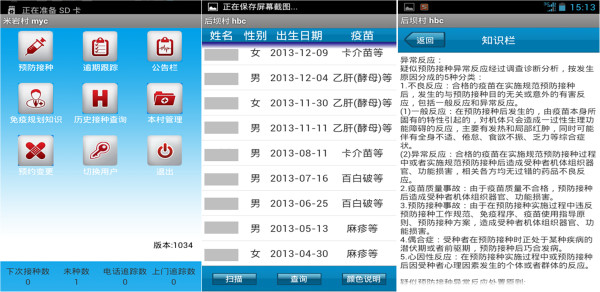
**Screenshot of the EPI app.** Legend: first screenshot shows the general function modules of EPI app. Second screenshot shows the list of scheduled vaccination appointments. The last screenshot shows the health education information.

1) Making appointments: the app will automatically push immunization information to each village doctor in the intervention arm. Once a child has established his/her immunization card, township doctors will enter the child’s general information and immunization information into the child EPI information system (computer platform). The system will automatically assign this information to corresponding village doctors and push child’s immunization information and scheduled vaccine dates to village doctors’ smart phone through this app (Figure 3).

2) Record: on the scheduled vaccine date, when a child comes to the village clinic and gets his/her vaccine, the village doctor will use the app to record a child’s immunization status (name of the vaccine, date, batch number, manufacturer, vaccine site and whether it is a free shot or not). The updated immunization information of a child will be updated to the local immunization management system automatically through the 3G wireless network.

3) Tracking: a list with the names of all children who missed their immunization appointment three times will be sent to village doctors in their catchment area. The name list also includes contact information (family phone number, cell phone number and address) of children’s caregivers. The village doctor will use this list to call children’s caregivers or drop by children’s homes to remind them about immunization.

4) Health education: program managers will tailor health information related to immunization such as the abnormal reactions, key knowledge and skills of intradermal and subcutaneous injections and send this to village doctors through this app. In this way, village doctors will learn knowledge about immunization at any time or any place.

#### Co-interventions and contamination

There are no known co-occurring health programs in this community that could potentially alter the outcomes. In addition, no other child health trials have ever been conducted in this community. To reduce the risk of contamination, we will assign each village doctor in the intervention arm a unique ID and password to log in the EPI app to avoid smart phone share between village doctors in the intervention arm and control arm.

### Control

1) Making appointments: when caregivers take their children to village clinics in control arm to receive vaccines, village doctors will schedule the next appointment for vaccination with caregivers. After that, when they manually update children’s information on the child EPI information system, the system will also generate a list on paper including the dates when children should have subsequent vaccinations. Then village doctors will bring this list as a reference for further appointments.

2) Record: on the scheduled vaccine date, when a child comes to the village clinic and gets his/her vaccine, the village doctor will record this child’s immunization status (name of the vaccine, date, batch number, manufacturer, vaccine site and whether it is a free shot or not) on paper. Then they will go to township hospitals to manually input children’s immunization information into the child EPI information system once a month.

3) Tracking: a name list with all overdue children who missed their immunization appointment will be manually generated from township hospitals monthly and then township hospital doctors will inform the village doctors by telephone calls.

4) Health education: village doctors will be trained at the township hospital during monthly regular meetings.

### Randomization

The unit of randomization will be the cluster, defined by the administrative boundary for each village. Randomization will be performed after the baseline household survey. A statistician outside the research team will be responsible for the randomization.

A short pre-randomization survey was conducted to explore the general characteristics of village doctors who provided immunization services in their catchment areas, as well as their mobile phone use preferences. There are 59 village doctors that provide immunization services in our study area.

For villages (n = 18) that have more than one village doctor who manages immunization, we will only include the youngest village doctor, because we assume younger doctors have better mobile phone use skills and can potentially increase the uptake and sustainability of the intervention. Four villages will be excluded because of the small number of children (<7 children aged 12–23 months) in these villages. One village will be randomly excluded because we use a paired design. In total, 36 villages (18 pairs) will be included for randomization.

Package of blockTools in R [17] will be used for matching and random allocation, for multiple continuous variables were allowed. We will use the number of children younger than 1 year managed by the village doctor in charge of vaccination injection, age of the youngest village doctor for immunization in village, and the population density (population/area in each village) for pair matching.

### Sample size and study population

A. Sample size calculation

Base on a survey conducted in early 2013 in Xuanhan County, Sichuang Province, the coverage of the five key vaccines is 41.35%. We expect that the five-vaccination coverage in intervention group will rise from 41.35% to 65%.With level of confidence of 95%, power of 80%, the coefficient of variation in outcome between clusters within matched pairs (Km) as 0.25 [18], we estimate that 7 participants from each cluster of 18 pairs of clusters will be sufficient to detect the difference in two groups.

B. Intervention target will be village doctor in charge of immunization

Inclusion criteria

a. Village doctors who are in charge of immunization (both management and injection)

b. If there are several village doctors available, we will choose the youngest one and with the assistant practicing license for clinical doctors

Exclusion criteria

a. Refuse to participate

b. The estimated number of children aged one to two years in village is less than 7. The estimated number is calculated as the number of children reported multiplied by 75% (the average percentage of children staying in the village) and 90% (the expected rate of contacted primary caregivers who agree and complete the survey).

C. The survey target will be caregivers of children aged 12–23 months. Children aged over 1 year are supposed to complete the five vaccinations required by national EPI program. Therefore, caregivers of children aged 12–23 month old are our survey target in both the baseline and endline survey.

Inclusion criteria

a. Living in Xuanhan County

b. Receiving immunization service in Xuanhan County

c. Having a child aged 12 to 23 months; if there is more than one child aged 12 to 23 months, only include the youngest one

d. Being a caregiver of the selected child

Exclusion criteria

a. Refuse to participate.

### Outcome measurement

The primary outcome is five-vaccine immunization coverage and the secondary outcome is immunization coverage for Bacillus Chalmette Guerin (BCG), Oral Poliomyelitis Vaccine (OPV), Diphtheria Pertussis Tetanus Combined Vaccine (DTP), Measles Vaccine (MV), Hepatitis B Vaccine (HBV), Hib vaccine, Rotavirus vaccine and Pneumococcal conjugate vaccine (PCV) vaccine (Additional file 1: Table S2).Immunization coverage will be collected by the immunization module (IM) of the household survey. The immunization card will be collected for every participant, and injection record including dosage and date will be recorded with *Goodata*, a smart phone app for data collection. The demographic information such as education level, ethnicity, gravidity, parity, type of Hukou, source of family income will also be collected by the household and identification module (HH and ID) in household survey, as they are factors that may potentially influence immunization.

### Multidimensional evaluation of smart phone app (EPI)

#### Heuristic evaluation

A heuristic evaluation is an informal usability evaluation technique to evaluate whether user interface elements of a system adhere to a set of usability principles known as heuristics [19]. The purpose of the heuristic evaluation method is to identify any serious usability problems that users might encounter and to recommend improvements to the design before implementing the system. By evaluating the interface in the developmental phase, design problems may be identified and corrected earlier, thus reducing potential usability errors [20].

Three experts who have experience in developing health information systems will be invited to conduct the heuristic evaluation. To make sure all the experts will receive the same guidance, materials including the user manual of EPI app, introduction of heuristic evaluation and heuristic evaluation form will be provided to them before evaluation starts. Research staff will make sure that the experts understand the procedure of evaluation and ask the experts to start using all four function modules of the app.

Violations are defined as problems that could potentially interfere with end user’s ability to interact effectively with the app [21]. After finishing with all function modules of the app, experts will fill out the heuristic evaluation form. During evaluation sessions, experts are encouraged to “think aloud”. Research staff will use a video camera to record the heuristic evaluation and take field notes. The time for completing each task and number of errors will also be recorded.

#### Usability test

Usability testing is a way of ensuring that apps are adapted to users and their tasks [22]. Usability testing involves measuring the performance of end users doing typical task case scenarios [22,23]. The goal of this fundamental step is to obtain objective performance data to assess an app in terms of usability goals: accuracy and acceptability. Accuracy will be measured by logging the time spent on completing each task and number of errors. Acceptability is defined as the extent of uptake of this app and will be assessed using users’ perception of ease of use and usefulness scale (PEUU) [24], which will be translated and adapted into Chinese context before use.

About 10–15 village doctors will be recruited for usability testing. The inclusion criteria for village doctors are: 1) in charge of immunization of children in his/her catchment area; 2) able to use a smart phone and apps; 3) do not receive any intervention in our study. In addition, we will exclude those who refuse to participate. Village doctors who meet our inclusion criteria will be invited to participate. After obtaining their informed consent, short training will be conducted to introduce how to use the smart phone and app as well as the basic function of app, usability testing, case scenarios and the PEUU scale assessment. Then, village doctors will be asked to use the app to perform case scenarios. Research staff will observe the whole process and record the time for completing each task and number of errors. Meanwhile, during evaluation sessions, village doctors are encouraged to “think aloud” on problems they encountered or experiences while using. After obtaining their consent, research staff will use video cameras to record the village doctors to perform case scenarios and take field notes. After completion of case scenarios, village doctors will fill out the PEUU scale and be encouraged to be as specific as possible.

#### Feasibility evaluation

Following usability testing, a feasibility evaluation will be conducted with village doctors in our intervention arm to determine compliance and satisfaction with the app. A questionnaire designed by the research team and a qualitative interview will be used to assess the user experience and satisfaction of village doctors. Technical or hardware problems encountered while using the app will also be collected during feasibility evaluation and regular monitoring and evaluation using both qualitative interviews and checklist form.

Village doctors in the intervention arm will be enrolled and will be given a smart phone with pre-installed app and asked to use the app to manage immunization in their catchment areas. Telephone assistance will be available to village doctors in case of technical problems. Every two months, research staff will call enrolled village doctors to assess the user experience and satisfaction when using the app with the self-designed questionnaire and semi-structured interview. Compliance, the reason for non-compliance, satisfaction, technical or hardware problem encountered while using the app and suggestions on improvement will be collected during feasibility evaluation and regular monitoring and evaluation field visits.

### Data management and analysis

The primary investigator will be in charge of the database maintenance and management. Data will be stored in a computer with passwords. The primary investigator will be responsible for the integrity and accuracy of the data. All participants will have a unique code. Smart phones will be used to collect data. Data will be secured first, then exported and uploaded to the internet into a database. Logic verification statements will be written in data acquisition software for the mobile phone. Statistical analyses will be performed by research staff blinded to the treatment allocation.

The principle of intention-to-treat-analysis will be used. At village level, the five-vaccine immunization coverage or other immunization coverage indicators will be transformed first and compared with paired t test or non-parametric test. Random-effect logistic regression will be used to test for the effect on every outcome when controlled for the covariate (socioeconomic variables such as education level, ethnicity, gravidity, parity, type of Hukou, source of family income) on individual level.

Exploratory statistical analysis of multidimensional evaluation of app will be used to calculate the frequencies and mean severity rating scores of heuristic violations, mean logging time, number of error for each task, score of perceived ease of use and usefulness, the percentage of compliance and frequency of technical or hardware problems. Qualitative materials of the multidimensional evaluation of app will be collected through recordings and evaluation forms. The recording will be transcribed verbatim and compared to field notes for any inconsistency or missing information. Content analysis will be used to analyze qualitative data.

All quantitative analysis will be performed by SAS 9.1 and qualitative analysis will be performed with MaxQDA. P < 0.05 will be considered statistically significant.

### Quality control

#### Quality control measures of surveys and interviews

Study supervisors will be recruited from Capital Institute of Pediatrics, who are trained and have experience with conducting household surveys. Interviewers will be trained for one day including the explanation of the questionnaire and how to collect data using a smart phone. Role play of a standard questionnaire case will be performed in the training course to assess inter- and intra-interviewer reliability for completing survey instruments after the training. The logic checking programs will be embedded into the data collection app. Quality-control measures also require that each supervisor attends interviews and will be responsible for identifying and solving problems on each survey day.

Qualitative interviewers will also be from Capital Institute of Pediatrics and are trained in qualitative interviewing and have had experience with conducting interviews in the field. All recording from qualitative interviews will be transcribed verbatim independently by two investigators. Comparison between two copies of transcription will be performed to check for inconsistencies. A third investigator will verify the transcripts by listening to the recording again. Then the transcripts will be imported into analytical software MaxQDA for analysis.

#### Quality control measure of intervention

The quality control of intervention will be conducted in the middle of the implementation and consists of two parts: qualitative monitoring and quantitative monitoring. Compliance, the reason for non-compliance, satisfaction, technical or hardware problems encountered while using the app and suggestions for improvement will be collected during the monitoring and evaluation visit.

### Ethical consideration and consent

The study protocol was approved by Ethics Committee of Capital Institute of Pediatrics (SHERLL 2013080). The research team will first communicate with staff in local health sector to make sure they understand the overall goal, objectives and implementation plan and to ask for approval of the study to take place in Xuanhan County. Consent for participation in the study will be obtained from caregivers who fulfill the inclusion criteria at the outset of the study. The trained interviewers will first read out the consent form to the participant and tell them if there are any problems, and they can refuse or quit if there is anything making them uncomfortable. At the end of the form, the participants will mark their agreement through a signature.

## Discussion

This paper describes the study protocol for a cluster randomized controlled trial of a smart phone app on child immunization. Given the existing gap in children immunization coverage in China, our study will provide important preliminary insights in using a smart phone app to improve child immunization in rural China and our study is unique in a number of ways.

First, to our knowledge, this is the first cluster randomized controlled trial of a mobile phone-based intervention using a smart phone app to support village doctors who work at primary level to manage childhood immunization. Given the limited evidence of effective interventions for childhood immunization in low- and middle- income settings, research in this area is in great need.

Second, our study examines the mobile health intervention’s effect on coverage of essential childhood vaccines included into national EPI using rigorous cluster randomized controlled trial design. Moreover, we will also evaluate intervention itself to explore its usability and feasibility, which will provide evidence for further scale-up. In addition with qualitative interviews, we will also know what app functions work and what does not work for village doctors.

However, our study also has several limitations. First, for villages that have multiple village doctors who are in charge of children immunization, we can only include the youngest one. Therefore, generalizability of the results of this trial to other settings with older doctors may be limited. We will select the youngest village doctor, because we think they have better technology capability and the sustainability of this intervention may be better. Second, the participants are not blinded. However, the outcome measures and the statistical analyses will be performed by research staff blinded to the treatment allocation.

This study will make a significant and unique contribution to our knowledge about using mHealth technology to improve immunization of children in rural China. The results will provide rigorous evidence to better inform government and health services provider on whether the smart phone app can aid better delivery of immunization services. This can be used to improve the currently low immunization coverage in rural Sichuan Province and other parts in China as well as similar population in other low- and middle-income settings.

## Abbreviations

mHealth: mobile health technology; apps: smart phone applications; MDGs: Millennium Development Goals; WHO: the World Health Organization; UNICEF: The United Nations Children’s Fund; BCG: Bacillus Chalmette Guerin; OPV: Oral Poliomyelitis Vaccine; DTP: Diphtheria Pertussis Tetanus Combined Vaccine; EPI: Expanded Program on Immunization; NHSS: National Health Service Survey; PDA: Portable Digital Assistant; C-RCT: cluster randomized controlled trial; CDC: Centers of Disease Control and Prevention; HBV: Hepatitis B Vaccine; MV: Measles Vaccine.

## Competing interests

The authors declare that they have no competing interests.

## Authors’ contributions

LC, YFZ, WW, XZD and XQR design the study and draft the manuscript. RKY and LZ from Save the Children China Program take charge of developing the intervention, contribute in conceptualizing the study and implementing the study. LC, WW, XZD, XQR, QW and YFZ will carry out the study. MV, YL and JCK made a substantial contribution to conception and design of the study and revised the article critically for important intellectual content. All authors contributed substantially in revising the manuscript, read and approved the final manuscript.

## Pre-publication history

The pre-publication history for this paper can be accessed here:

http://www.biomedcentral.com/1471-2458/14/262/prepub

## Supplementary Material

Additional file 1: Table S1 Basic child immunization program in China. **Table S2** the immunization coverage indicators. Description: the immunization process in China and the coverage indicators for immunization. Click here for file
