# Supplementary material for: Effectiveness of a smart phone app on improving immunization of children in rural Sichuan Province, China: study protocol for a paired cluster randomized controlled trial
Source: BMC Public Health. 2014 Mar 20;14:262. doi: 10.1186/1471-2458-14-262 (PMC3999920; doi:10.1186/1471-2458-14-262)
Supplement: Additional file 1: Table S1 — Basic child immunization program in China. Table S2 the immunization coverage indicators. Description: the immunization process in China and the coverage indicators for immunization. [file 1471-2458-14-262-S1.docx]

Additional file 1: Table S1 Basic child immunization program in China

| Age in  Months | BCG | OPV | DPT | MV | HBV |
| --- | --- | --- | --- | --- | --- |
| 0 | Dose 1 |  |  |  | Dose 1 |
| 1 |  |  |  |  | Dose 2 |
| 2 |  | Dose 1 |  |  |  |
| 3 |  | Dose 2 | Dose 1 |  |  |
| 4 |  | Dose 3 | Dose 2 |  |  |
| 5 |  |  | Dose 3 |  |  |
| 6 |  |  |  |  | Dose 3 |
| 8 |  |  |  | Dose 1 |  |
| 18-24 |  |  | Dose 4 |  |  |
| 48 |  | Dose 4 |  |  |  |

BCG: Bacillus Chalmette Guerin

OPV: Oral Poliomyelitis Vaccine

DTP :Diphtheria Pertussis Tetanus Combined Vaccine

MV: Measles Vaccine

HBV: Hepatitis B Vaccine

Additional file 1: Table S2 The immunization coverage indicators

| Vaccine | Immunization time (month) | | Number of doses for children under 1 year | | Indicator | Definition | Numerator | Denominator |
| --- | --- | --- | --- | --- | --- | --- | --- | --- |
| Hepatitis B (HepB) | 0、1、6 | | 3 | | Hepatitis B immunization coverage | Percentage of children aged 12-23 months immunized with 3 doses of hepatitis B vaccine | Surveyed children of 12-23 months who were immunized with 3 doses of hepatitis B vaccine | All surveyed children of 12-23 months |
| BCG | At birth | | 1 | | BCG immunization coverage | Percentage of children aged 12-23 months immunized with BCG vaccine | Surveyed children of 12-23 months who were immunized with 1 dose of BCG vaccine | All surveyed children of 12-23 months |
| OPV | 2、3、4 | | 3 | | OPV immunization coverage | Percentage of children aged 12-23 months immunized with 3 doses of OPV vaccine | Surveyed children of 12-23 months who were immunized with 3 doses of OPV vaccine | All surveyed children of 12-23 months |
| DPT | 3、4、5 | | 3 | | DPT immunization coverage | Percentage of children aged 12-23 months immunized with 3 doses of DPT vaccine | Surveyed children of 12-23 months who were immunized with 3 doses of DPT vaccine | All surveyed children of 12-23 months |
| Measles (MV) | 8 | | 1 | | Measles immunization coverage | Percentage of children aged 12-23 months immunized with measles-containing vaccine | Surveyed children of 12-23 months who were immunized with measles vaccine | All surveyed children of 12-23 months |
| Five-vaccine immunization | | | | | Five-vaccine immunization coverage | Percentage of children aged 12-23 months immunized with HepB, BCG, OPV, DPT and measles vaccines | Surveyed children of 12-23 months who were immunized with all doses of five vaccines that they should be given before 1 year old | All surveyed children of 12-23 months |
| Hib | | Not specified | | 2~3 | Hibimmunization coverage | Percentage of children aged 12-23 months immunized with Hib vaccine | Surveyed children of 12-23 months who were immunized with at least 1 dose of Hib vaccine | All surveyed children of 12-23 months |
| Rotavirus | | Not specified | | 1 | Rotavirus immunization coverage | Percentage of children aged 12-23 months immunized with rotavirus vaccine | Surveyed children of 12-23 months who were immunized with 1 dose of Rotavirus vaccine | All surveyed children of 12-23 months |
| PCV | | Not specified | | 3 | PCV immunization coverage | Percentage of children aged 12-23 months immunized with PCV vaccine | Surveyed children of 12-23 months who were immunized with at least 1 dose of PCV vaccine | All surveyed children of 12-23 months |
